# Predicting Antigenic Variants of Influenza A/H3N2 Viruses

**DOI:** 10.3201/eid1008.040107

**Published:** 2004-08

**Authors:** Min-Shi Lee, Jack Si-En Chen

**Affiliations:** * MedImmune Vaccines, Mountain View, California, USA

**Keywords:** influenza, antigenicity, vaccine strain, hemagglutinin, prediction model, antigenic variants, bioinformatics, research

## Abstract

Current inactivated influenza vaccines provide protection when vaccine antigens and circulating viruses share a high degree of similarity in hemagglutinin protein. Five antigenic sites in the hemagglutinin protein have been proposed, and 131 amino acid positions have been identified in the five antigenic sites. In addition, 20, 18, and 32 amino acid positions in the hemagglutinin protein have been identified as mouse monoclonal antibody–binding sites, positively selected codons, and substantially diverse codons, respectively. We investigated these amino acid positions for predicting antigenic variants of influenza A/H3N2 viruses in ferrets. Results indicate that the model based on the number of amino acid changes in the five antigenic sites is best for predicting antigenic variants (agreement = 83%). The methods described in this study could be applied to predict vaccine-induced cross-reactive antibody responses in humans, which may further improve the selection of vaccine strains.

## Abstract 2

Models based on amino acid changes in influenza hemagglutinin protein were compared to predict antigenic variants of influenza A/H3N2 viruses.

Influenza viruses cause substantial medical and social problems throughout the world, and vaccination is the primary method for preventing influenza and its complications. Of the three types of influenza viruses (A, B, and C), only influenza A and B viruses cause epidemic human disease. Hemagglutinin (HA) and neuraminidase proteins are the two surface antigens that induce protective antibody responses and are the basis for subtyping influenza A viruses. Influenza B viruses are not categorized into subtypes (1). Since 1977, influenza A/H1N1, A/H3N2, and B viruses have been in global circulation, and these three viruses are currently included as vaccine components. Current inactivated vaccines provide essential protection when the vaccine antigens and the circulating viruses share high degree of similarity in the HA protein. Since new influenza virus antigenic variants emerge frequently from accumulation of point mutations in the HA protein (i.e., antigenic drift), influenza vaccine antigens need to be updated frequently, based on the results of global influenza surveillance (1), which includes clinical, virologic, and immunologic surveillance. In virologic surveillance, influenza viruses are characterized antigenically on the basis of ferret serum antibody cross-reactivity. Antigenic variants selected serologically are then tested for antibody cross-reactivity in human sera to evaluate the potential cross-protection against the antigenic variants provided by the current vaccines and to select vaccine strains for the next season (2,3).

The HA protein of influenza viruses is synthesized as a single polypeptide (HA0) that is subsequently cleaved into two polypeptides (HA1 and HA2) and forms into homotrimers. The HA1 polypeptide mutates more frequently than the HA2 polypeptide and plays a major role in natural selection (4,5). Three-dimensional (3-D) structure of the HA protein of A/Aichi/2/68 (H3N2) has been determined, and five antigenic sites on the HA1 polypeptide have been proposed conceptually (4–6). Of the 329 amino acid positions on HA1, 131 lie on or near the five antigenic sites (7,8). Twenty amino acid positions on HA1 have been mapped, based on laboratory variants selected in the presence of mouse monoclonal antibodies (9,10). In addition, 18 amino acid positions have been identified as being under positive selection by comparing 357 viruses isolated from 1984 to 1996 (7). In a recent study, 32 amino acid positions have been identified as diverse codons by comparing 525 viruses isolated from 1968 to 2000 (11). However, the importance of these amino acid positions in terms of predicting antibody cross-reactivity is unclear. Therefore, we conducted this study to explore the usefulness of these amino acid positions for predicting antigenic variants of influenza A/H3N2 viruses. The methods described in this study could be used to predict vaccine-induced cross-reactive antibody responses in humans, which may further improve the selection of vaccine strains.

## Methods

### Cross-Reactive Antibody Data

In the current global influenza surveillance system, influenza viruses are characterized antigenically based on ferret serum hemagglutinin-inhibition (HAI) antibody cross-reactivity. We first screened publications for influenza H3N2 virus cross-reactive antibody data. Then, we searched the H3N2 viruses with cross-reactive antibody data for their amino acid sequences of the HA1 polypeptide (www.flu.lanl.gov) (8). Table 1 shows the full name, abbreviation, identification (ID) by type, and accession code of the H3N2 viruses (12–16). Six sets of ferret serum HAI cross-reactivity data were available for analysis. The first set included 11 viruses (55 pairwise comparisons, virus ID: A to K) isolated from 1971 to 1979 (12). The second set included 8 viruses (28 pairwise comparisons, virus ID: J, L to R) isolated from 1979 to 1987 (17). The third set included 10 viruses (45 pairwise comparisons, virus ID: S to AB) isolated from 1989 to 1994 (13). The fourth set included 8 viruses (28 pairwise comparisons, virus ID: AC to AJ) isolated from 1994 to 1996 (18). The fifth set included 5 viruses (10 pairwise comparisons, virus ID: AE, AK to AN) isolated from 1995 to 1999 (15). The sixth set included 6 viruses (15 pairwise comparisons, virus ID: AN to AT) isolated from 1999 to 2002 (16). A mathematical method had been proposed to calculate "antigenic relatedness" between two viruses (presented as a percentage) as a geometric mean of two ratios between the heterologous and homologous antibody titers (19,20).

**Table 1:** Full name, identification (ID), abbreviation, and accession code of influenza H3N2 viruses

Full name	ID	Abbreviation	Accession no.
A/Hong Kong/107/71	A	HK71	ISDNHK71
A/England/42/72	B	ENG72	ISDNENG72
A/Port Chalmers/1/73	C	PC73	ISDNPC73
A/Mayo Clinic/1/75	D	MC75	ISDNMC75
A/Victoria/3/75	E	VIC75	ISDNVIC75
A/Tokyo/1/75	F	TOK75	ISDNTOK75
A/England/864/75	G	ENG75	ISDNENG75
A/Allegheny County/29/76	H	AC76	Direct entry (12)
A/Victoria/112/76	I	VIC76	Direct entry (12)
A/Bangkok/1/79	J	BAN179	ISDNBK179
A/Bangkok/2/79	K	BAN279	ISDNBK279
A/Philippines/2/82	L	PHI82	ISDNPH282
A/Mississippi/1/85	M	MIS85	AF008893
A/Leningrad/360/86	N	LEN86	AF008903
A/Shanghai/11/87	O	SHA87	AF008886
A/Sichuan/2/87	P	SIC87	AF008884
A/Sydney/1/87	Q	SYD87	AF008882
A/Victoria/7/87	R	VIC87	AF008888
A/Beijing/353/89	S	BEI89	Z46391
A/Hong Kong/34/90	T	HK90	Z46409
A/Beijing/32/92	U	BEI92	Direct entry (13)
A/Hong Kong/23/92	V	HK92	Direct entry (13)
A/Guangdong/25/93	W	GUA93	Z46406
A/Madrid/252/93	X	MAD93	Z46411
A/Scotland/142/93	Y	SCO142	Z46413
A/Scotland/160/93	Z	SCO160	Z46414
A/Shangdong/9/93	AA	SHA93	Z46417
A/Hong Kong/1/94	AB	HK94	Z46407
A/Johannesburg/33/94	AC	JOH94	AF008774
A/Alaska/10/95	AD	ALA95	AF008748
A/Nanchang/933/95	AE	NCH95	AF008725
A/Wuhan/359/95	AF	WHN95	AF008722
A/Auckland/5/96	AG	AUC96	AF008714
A/Fujian/47/96	AH	FUJ96	AF008726
A/New York/37/96	AI	NY96	AF180650
A/South Africa/1147/96	AJ	SA96	Direct entry (14)
A/Sydney/5/97	AK	SYD97	SDNASYD97
A/Ireland/10586/99	AL	IRE99	Direct entry (15)
A/Moscow/10/99	AM	MOS99	ISDN13277
A/Panama/2007/99	AN	PAN99	ISDNCDA001
A/Fujian/140/2000	AO	FUJ00	Direct entry (16)
A/Chile/6416/2001	AP	CHI01	Direct entry (16)
A/New York/55/2001	AQ	NY01	Direct entry (16)
A/Fujian/411/2002	AR	FUJ02	ISDN38157
A/Hong Kong/1550/2002	AT	HK02	Direct entry (16)

Since our study investigates the relationship between antigenic difference and amino acid changes in the HA1 polypeptide, the mathematical method was modified to calculate "antigenic distance" (i.e., reciprocal of antigenic relatedness). For example, if homologous titers of two viruses are 640 and 640 and two heterologous titers against each other are 320 and 320, the antigenic relatedness between these two viruses is ([320 x 320]/[640 x 640])^½^ = 50%, and the antigenic distance between these two viruses is ([640 x 640]/[320 x 320])^½^ = 2. Table 2 shows the antigenic distances of the 55 pairwise comparisons among the 11 viruses in the first set. In total, 181 pairwise comparisons among 45 viruses were available for analysis. Among the 181 pairwise comparisons, 56 (31%) have an antigenic distance <4 (i.e., similar antigenicity), and 125 (69%) have an antigenic distance >4 (i.e., antigenic variant) (21).

**Table 2:** Antigenic distance (upper right) and number of amino acid changes in the HA1 (lower left) in 55 pairwise comparisons among 11 influenza H3N2 viruses

Virus ID^a^	H3N2 virus																				
	A	B	C	D	E	F	G	H	I	J	K										
A. HK/71		27.7	19.6	39.2	55.4	39.2	48.0	39.2	110.9	67.9	110.9										
B. ENG/72	15		4.0	26.1	16.0	64.0	156.8	4.0	64.0	78.4	181.0										
C. PC/73	16	7		8.0	16.0	32.0	27.7	22.6	37.0	55.4	90.5										
D. MC/75	21	12	12		9.2	32.0	45.3	32.0	90.5	55.4	90.5										
E. VIC/75	30	19	19	15		11.3	27.7	1.9	5.7	78.4	128.0										
F. TOK/75	20	17	18	16	20		78.4	45.3	26.1	39.2	90.5										
G. ENG/75	27	18	17	8	17	22		32.0	4.6	6.9	19.6										
H. AC/76	31	20	18	16	6	21	19		9.2	78.4	73.9										
I. VIC/76	32	21	19	17	2	22	19	4		27.7	32.0										
J. BAN/1/79	36	25	23	21	24	33	17	26	26		9.2										
K. BAN/2/79	36	24	24	22	26	33	20	28	28	3											

^a^ID, identification.

### Sequence Alignment

Amino acid sequences of the HA1 polypeptide were downloaded from the Los Alamos Influenza Sequence Database (8) or entered from the original publications if they were not available from the Los Alamos Influenza Sequence Database. Amino acid sequences of the 45 viruses were harmonized to same length (329 residues) and were numbered according to A/Aichi/2/68 HA1 sequence because the 3-D structure of the A/Aichi/2/68 hemagglutinin protein has been determined (4–6). Pairwise alignments among the 45 sequences were conducted by using S-Plus 2000 (Insightful Corporation, Seattle, WA). Pairwise-aligned amino acid sequence data were transformed into 0 (without change) and 1 (with change) and were further linked with the pairwise antigenic distance data for predicting analyses.

### Predicting Antigenic Variants

The first model was based on amino acid differences in the whole HA1 polypeptide (329 residues). The second model was based on amino acid differences in the five antigenic sites (131 residues) (Appendix) (7,8). The third model was based on the 20 positions related to mouse monoclonal antibody binding (Appendix) (9,10). The fourth model was based on the 18 positions under positive selection (Appendix) (7). The fifth model was based on the 32 codons of substantial diversity (Appendix) (11). For evaluating the qualitative performance of the five prediction models, an antigenic variant was defined as antigenic distance >4 (21). Positive predictive value (PPV), negative predictive value (NPV), and agreement of the five prediction models were calculated, and different cutoff levels of amino acid differences were compared by using the receiver-operating characteristic analysis (22).

## Results

### Model One

Figure A shows the scatterplot between antigenic distance and number of amino acid changes in the HA1 peptide (328 residues). Among the 181 pairwise comparisons, the antigenic distance ranged from 1 to 181, and the number of amino acid changes in the HA1 peptide ranged from 1 to 36. Overall, the antigenic distance correlated to the number of amino acid changes in the HA1 polypeptide (R = 0.74, p < 0.001). Different cutoffs of amino acid changes in the HA1 polypeptide were evaluated for predicting antigenic variants. The highest agreement was found with a cutoff of >7 amino acid changes, which shows that the NPV, PPV, and agreement were 66% (31/47), 81% (109/134), and 77% (140/181), respectively (Figure A).

**Figure:**
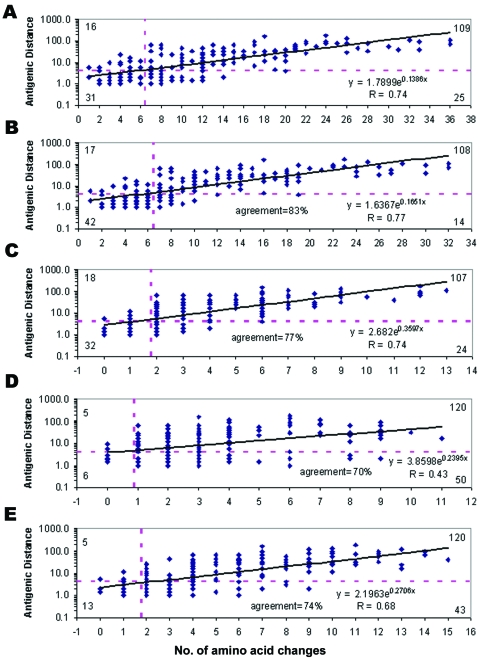
Performance of the five prediction models. Solid line at each plot, regression; horizontal dashed line, cutoff of antigenic distance >4; vertical dashed line, cutoff of number of amino acid changes. Numbers at the four corners indicate true negative (lower left), false negative (upper left), true positive (upper right), false positive (lower right) in each prediction model. A) The first model was based on amino acid differences in the whole HA1 polypeptide (329 residues). B) The second model was based on amino acid differences in the five antigenic sites (131 residues). C) The third model was based on the 20 positions related to mouse monoclonal antibody binding. D) The fourth model was based on the 18 positions under positive selection. E) The fifth model was based on the 32 codons with substantial diversity.

Table 3 shows some unique pairwise comparisons with unusual patterns between antigenic distances and amino acid changes. A/Shanghai/11/87 and A/Victoria/7/87 were antigenically different (antigenic distance = 5.7), but they had only one amino acid difference (R247S). The position 247 is located at the antigenic site D. In addition to the amino acid change at position 247, A/Shanghai/11/87 had two more amino acid differences from A/Sichuan/2/87 (E156K, S186V) and A/Sydney/1/87 (A138S, N193K), but these three viruses were antigenically similar (antigenic distance <4). A/Victoria/7/87 had only two amino acid differences from A/Sichuan/2/87 (K156E, V186S) and A/Sydney/1/87 (S138A, K193N), but A/Victoria/7/87 was antigenically different from these two viruses (Table 3). The positions 156, 186, and 193 are located at the antigenic site B and the position 138 is located at the antigenic site A. Moreover, the positions 156 and 193 are also located at the mouse monoclonal antibody-binding sites (Table A1).

**Table 3:** Some unique pairwise comparisons showing antigenic distance and amino acid changes

Viruses compared	Antigenic distance (ferret HAI titers)^a^	Amino acid changes (antigenic sites)
A/Shanghai/11/87 vs. A/Victoria/7/87	5.7 ([320 x 320]/[40 x 80])^½^	R247S(D)
A/Shanghai/11/87 vs. A/Sichuan/2/87	2.8 ([320 x 640]/[160 x 160])^½^	E156K(B), S186V(B), R247S(D)
A/Shanghai/11/87 vs. A/Sydney/1/87	2.0 ([320 x 320]/[160x 160])^½^	A138S(A), N193K(B), R247S(D)
A/Sichuan/2/87 vs. A/Victoria/7/87	5.7 ([320 x 640]/[40 x 160])^½^	K156E(B),V186S(B)
A/Sydney/1/87 vs. A/Victoria/7/87	4.0 ([320 x 320]/[80 x 80])^½^	S138A(A), K193N(B)
A/Victoria/3/75 vs. A/Victoria/112/76	5.7 ([640 x 2,560]/[640 x 80]) ^½^	L3F, R229G(D)
A/Sydney/5/97 vs. A/Panama/2007/99	1.4([5,120x 2,560]/[2,560 x 2,560])^½^	I3L, P21S, R57Q(E), Y137S(A), S142R(A), I144N(A), D172E(D), H183L, T192I(B), I194L(B), I226V(D), H233Y
A/Fujian/140/2000 vs. A/HK/1550/2002	2.0 ([640 x 640]/[320 x 320])^½^	G14C, A43V, R50G(C), E83K(E), N96S(D), S186V(B), V194I(B), P199S, V202I, W222R, G225D, I226V(D), C247S(D), S273P(C)
A/Chile/6416/01 vs. A/HK/1550/02	2.0 ([320 x 640]/[80 x 640])^½^	R50G(C), E83K(E), N96S(D), V106A, D144N(A), G186V(B), L194I(B), V202I, H221P, W222R, G225D, K246N(D)

^a^Hemagglutinin-inhibition (HAI) titers were shown as two homologous titers divided by two heterologous titers.

The unusual patterns between antigenic distances and amino acid differences may be due to interaction between amino acid changes in the hemagglutinin or laboratory variability, which needs further experiments to clarify. In addition, A/Victoria/3/75 and A/Victoria/112/76 had only two amino acid differences (L3F, R229G), but they were antigenically different (antigenic distance = 5.7) (Table 3), which also requires further experiments to clarify. The position 3 is not located at any antigenic site, and the position 229 is located at the antigenic site D. We found that 3 of 80 pairwise comparisons with >12 amino acid changes had antigenic distance <4 (Figure A).

A/Sydney/5/97 and A/Panama/2007/99 had 12 amino acid differences, but these two viruses were antigenically similar (antigenic distance = 1.4) based on ferret serum HAI titers (Table 3). However, inactivated vaccines containing A/Sydney/5/97 induced low serum antibody titers against A/Panama/2007/99 in humans; therefore, A/Sydney/5/97 was replaced by A/Panama/2007/99 as the vaccine strain for the 2000–01 season (3). A/HK/1550/2002 had 12 amino acid differences from A/Chile/6416/01 and 14 amino acid differences from A/Fujian/140/2000, but A/HK/1550/2002 was antigenically similar to these two viruses (Table 3). These three comparisons may indicate that interaction of multiple amino acid changes could potentially preserve the 3-D structure of HA1. Alternatively, the ferret serum HAI assay system is not sensitive enough to detect the antigenic difference.

### Model Two

Figure B shows the scatterplot between antigenic distance and number of amino acid changes in the five antigenic sites (131 amino acid positions). Among the 181 pairwise comparisons, amino acid changes in the five antigenic sites ranged from 1 to 32. Overall, the antigenic distance correlated to number of amino acid changes in the five antigenic sites (R = 0.77, p < 0.001). Different cutoffs of amino acid changes in the five antigenic sites were evaluated for predicting antigenic variants. The highest agreement was found by using a cutoff of >7 amino acid changes, which shows that the NPV was 71% (42/59), PPV was 89% (108/122), and agreement was 83% (150/181) (Figure B).

### Model Three

Figure C shows the scatter plot between antigenic distance and number of amino acid changes in the 20 amino acid positions related to mouse monoclonal antibody binding. Overall, the antigenic distance correlated to number of amino acid changes in the 20 amino acid positions (R = 0.74, p < 0.001). Different cutoffs of amino acid changes in the previously defined 20 amino acid positions were evaluated for predicting antigenic variants. The highest agreement was found by using a cutoff of >2 amino acid changes, which shows that the NPV was 64% (32/50), PPV was 82% (107/131), and agreement was 77% (139/181) (Figure C).

### Model Four

Figure D shows the scatterplot between antigenic distance and number of amino acid changes in the 18 amino acid positions under positive selection. Overall, the antigenic distance correlated moderately to number of amino acid changes in the 18 amino acid positions (R = 0.43, p < 0.001). Different cutoffs of amino acid changes in the 18 amino acid positions were evaluated for predicting antigenic variants. The highest agreement was found by using a cutoff of >1 amino acid changes, which shows that the NPV was 55% (6/11), PPV was 71% (120/170), and agreement was 70% (126/181) (Figure D).

### Model Five

Figure E shows the scatter plot between antigenic distance and number of amino acid changes in the 32 codons with substantial diversity. Overall, the antigenic distance correlated moderately to number of amino acid changes in the 32 codons (R = 0.68, p < 0.001). Different cutoffs of amino acid changes in the 32 codons were evaluated for predicting antigenic variants. The highest agreement was found by using a cutoff of >2 amino acid changes, which shows that the NPV was 72% (13/18), PPV was 74% (120/163), and agreement was 74% (133/181) (Figure E). Overall, the model based on the number of amino acid changes in the five antigenic sites has the highest correlation to the antigenic distance (R = 0.77) and the best performance for predicting antigenic variants (agreement = 83%).

## Discussion

Wilson and Cox proposed that a drift variant of epidemiologic importance usually contains >4 amino acid changes located on >2 of the five antigenic sites, but they did not specify the amino acid positions in the five antigenic sites (5). Our study further showed that the model based on the number of amino acid changes in the 131 amino acid positions in the five antigenic sites had the highest correlation to the antigenic distance and the best performance for predicting antigenic variants. Theoretically, not all 131 amino acid positions in the five antigenic sites play a critical role in determining antigenicity, and some immunodominant positions (i.e., major antibody-binding sites) could be identified by using bioinformatics models and reverse genetic techniques (23–25). A model based on the immunodominant positions can potentially have a better performance than the model based on the five antigenic sites.

The model based on the 20 amino acid positions related to mouse monoclonal antibody binding only have moderate performance for predicting antigenic variants (R = 0.74, agreement = 77%), which indicates that mouse and ferret antibodies may recognize different B-cell epitopes. In addition, that models four and five have a low performance for predicting antigenic variants is not surprising, since these two models identified the amino acid positions only on the basis of virus sequence data without incorporating antigenic properties.

Antigenic variants of influenza viruses are currently determined with the ferret serum HAI assay. The ferret serum HAI assay works well to distinguish major drift variants, but moderate differences are difficult to define reliably (26). As shown in Table 3, some unusual patterns between antigenic distance and amino acid changes in the HA1 may be caused by laboratory variability of the ferret serum HAI assay. The prediction models proposed in the present study may perform better if a more reliable assay system is used. Several studies have shown that neutralization assays are more sensitive for detecting influenza virus antibody responses than HAI assays (27,28). However, traditional neutralization assays based on cytopathic effect are labor-intensive and not suitable for a large-scale surveillance system. A simplified EIA-based neutralization assay may be the potential solution (29).

Several studies have documented that one to three amino acid changes in the HA1 of influenza H1N1 and H3N2 viruses could possibly reduce the antigenicity and efficacy of inactivated vaccines in animal models (30–33), which are consistent with our results (Table 3). In animal studies, single mutation at amino acid position 156 of the HA1 of two H3N2 viruses was linked to the reduced antigenicity (32,33). The position 156 is located at the antigenic site B and the mouse monoclonal antibody-binding site (Table A1). Overall, this evidence may indicate the existence of immunodominant positions in the HA1 and emphasize the importance of identifying the immunodominant positions to monitor the selection of vaccine strains and the process of vaccine manufacturing.

The current global surveillance system largely relies on ferret serum HAI data for selection of influenza vaccine strains (2,3). In some cases, human and ferret cross-reactive antibody data were not consistent (34,35). The methods described in this study could be applied to predict vaccine-induced cross-reactive antibody responses in humans, which may further improve the selection of vaccine strains (35).

## Appendix

**Table A1:** Amino acid positions compared in this study^a,b^

aa	Antigenic sites	MAb-20	PS-18	D-32	aa	Antigenic sites	MAb-20	PS-18	D-32
2				X	172	D			X
3				X	173	D			
5				X	174	D			
31				X	175	D			
44	C				176	D			
45	C				177	D			
46	C				179	D			
47	C				182	D			
48	C				186	B		X	
50	C				187	B			
51	C				188	B	X		
53	C	X			189	B	X		X
54	C				190	B		X	X
57	E				192	B			
59	E				193	B	X	X	X
62	E	X		X	194	B		X	
63	E	X			196	B			X
67	E				197	B		X	X
75	E				198	B	X		
78	E				201	D		X	
80	E				203	D			
81	E				205		X		
82	E				207	D			
83	E			X	208	D			
86	E				209	D			
87	E				212	D			
88	E				213	D			
91	E				214	D			
92	E				215	D			
94	E				216	D			
96	D				217	D			
102	D				218	D	X		
103	D				219	D			
109	E				220				X
112				X	226	D		X	X
117	D				227	D			
121	D		X	X	228	D			
122	A				229	D			
124	A		X	X	230	D			
126	A				238	D			
128	B				240	D			
129	B				242	D			
130	A				244	D			
131	A			X	246	D			
132	A				247	D			
133	A	X	X	X	248	D			
135	A	X	X	X	260	E			
137	A				261	E			
138	A		X		262	E		X	X
140	A				265	E			
142	A	X	X	X	271				X
143	A	X			273	C			
144	A	X		X	275	C		X	X
145	A	X	X	X	276	C			X
146	A	X			278	C			X
150	A				279	C			
152	A				280	C			
155	B				294	C			
156	B	X	X	X	297	C			
157	B	X		X	299	C			X
158	B	X	X	X	300	C			
159	B				304	C			
160	B				305	C			
163	B				307	C			
164	B				308	C			
165	B	X			309	C			
167	D				310	C			
168	A				311	C			
170	D				312	C			
171	D								

^a^aa, amino acid; MAb, monoclonal antibody. ^b^Among the 329 amino acid residues in the HA1 polypeptide, 131, 20, 18, and 32 amino acid positions have been identified as in the five antigenic sites (A, B, C, D, E), the mouse monoclonal antibody-binding sites (MAb-20), the positively selected codons (PS-18), and the codons with substantial diversity (D-32), respectively.
